# Environmental health literacy among oral healthcare professionals—a comparison across professional groups

**DOI:** 10.3389/fpubh.2026.1933350

**Published:** 2026-10-19

**Authors:** Reem Al-Kattan

**Affiliations:** Department of Periodontics and Community Dentistry, King Saud University, Riyadh, Saudi Arabia

**Keywords:** dentists, environmental health literacy, oral health professionals, Saudi Arabia, sustainable healthcare

## Abstract

**Introduction:**

The present study aimed to assess environmental health literacy (EHL) among oral healthcare professionals and compare levels between dentists and non-dentist personnel in oral healthcare settings in Saudi Arabia.

**Methods:**

The study included dentists and non-dentist oral healthcare professionals (academicians, dental assistants, and allied health professionals) recruited using a convenience sampling approach. Data was collected using a structured questionnaire assessing four EHL domains: knowledge, attitude, confidence, and practice, along with demographic and professional characteristics. Responses were recorded on a 5-point Likert scale, and composite scores were calculated, with ≥70% considered a good literacy level. Data was analyzed using R Statistical Software, employing descriptive statistics, chi-square and t-tests, Spearman correlation, and multivariable logistic regression analyses.

**Results:**

A total of 384 participants were included, of whom 258 were dentists and 126 were non-dentists. The overall literacy mean (SD) scores were comparable between dentists and non-dentists, with no statistically significant difference in overall literacy levels, at 70.5 (15.1) and 73.4 (15.8), respectively (*p* = 0.090). However, non-dentists demonstrated significantly higher knowledge scores and a greater proportion of good knowledge levels compared with dentists (*p* < 0.05), while no significant differences were observed in attitude, confidence, or practice domains. Significant positive correlations were found among all literacy domains in both groups. Regression analysis showed that among dentists, older age, attendance at environmental health events, and prior training were significantly associated with higher literacy levels, whereas among non-dentists, attendance at scientific events and place of residence were significant predictors.

**Conclusion:**

Overall, EHL was moderate among oral healthcare professionals, with notable differences in knowledge favoring non-dentists. Strengthening environmental health education and continuing professional development is essential to enhance literacy levels and promote sustainable oral healthcare practices.

## 1 Introduction

Environmental health has emerged as a major public health priority due to the increasing burden of environmentally associated diseases and the growing recognition of environmental determinants of health. Climate change, air pollution, hazardous waste exposure, occupational contaminants, and unsustainable healthcare practices collectively contribute to adverse human health outcomes and healthcare system challenges (1, 2). Healthcare professionals play a vital role in addressing these effects through environmentally responsible clinical practice, patient education, and the implementation of sustainable healthcare strategies. Within dentistry, environmental sustainability has emerged as an increasingly important component of oral healthcare, with growing attention to sustainable clinical practices, waste management, and incorporation of environmental sustainability into oral health professional education (3).

Environmental health literacy (EHL) refers to the ability to access, understand, evaluate, and apply information related to environmental health risks and preventive behaviors. Adequate EHL enables healthcare professionals to recognize environmentally associated health conditions, communicate environmental risks effectively, and integrate preventive measures into clinical practice. Despite the expanding global emphasis on sustainability and environmental responsibility in healthcare systems, evidence suggests that environmental health competencies remain insufficiently integrated into healthcare education and professional training (4–6).

Dental healthcare settings are uniquely associated with environmental concerns due to the extensive use of chemical agents, biomedical waste generation, radiation exposure, water consumption, and infection control materials. Therefore, oral healthcare professionals are expected to possess sufficient environmental health awareness and competencies to support environmentally responsible clinical practice (7, 8). Previous studies assessing environmental health awareness among healthcare professionals have primarily focused on medical practitioners, nursing professionals, or public health personnel, with relatively little attention directed toward oral healthcare teams (9, 10). Understanding the current status of EHL among oral healthcare professionals is essential for identifying educational gaps and informing curriculum development, continuing professional education, and policy initiatives. The aim of the current study was to assess and compare EHL levels among dentists and non-dentist oral healthcare professionals. The study also explored associations between demographic and professional factors and good literacy levels within both groups.

## 2 Materials and methods

This cross-sectional study was conducted to assess EHL among oral healthcare professionals. The study included dentists and non-dentist oral healthcare personnel, including academicians, dental assistants, and allied health professionals. Participants were recruited using a convenience sampling via email from dental healthcare and academic settings in Saudi Arabia.

Eligible participants were adults currently involved in oral healthcare-related practice or academic activities. Participation was voluntary, and informed consent was obtained electronically before survey completion. The study adhered to ethical principles governing human participant research and ethical approval was obtained from the Institutional Review Board of King Saud University Medical City (Project no. E-25-9816).

EHL was evaluated using a structured researcher-developed self-administered questionnaire consisting of multiple domains related to environmental health. The survey also collected demographic and professional information, including age, gender, educational level, nationality, residence, prior environmental health training, and attendance at environmental health scientific events (Supplementary Table S1).

The questionnaire was developed based on a review of the relevant literature on environmental health and EHL, with consideration of the roles and responsibilities of oral healthcare professionals. The research team generated items to assess four domains of EHL: knowledge, attitudes, confidence, and practices related to environmental health. The initial questionnaire was reviewed by members of the research team for content relevance, clarity, and appropriateness to the target population, and items were revised accordingly before administration.

EHL was conceptualized as a multidimensional construct encompassing knowledge, attitudes, confidence, and practices related to environmental health. These domains were selected to capture complementary aspects of EHL among oral healthcare professionals, from awareness and understanding of environmental risks to perceived ability and actual engagement in addressing such risks. The questionnaire was contextualized to oral healthcare by incorporating environmental issues relevant to dental practice, including the environmental impacts of dental facilities and materials, sustainability practices, energy and water use, waste reduction, and environmental health communication with patients.

The questionnaire was pilot tested among a small group of oral healthcare professionals who were not included in the final study sample to assess the clarity, comprehensibility, and feasibility of the questionnaire. Feedback obtained during the pilot testing was used to refine the wording and presentation of the items. Content validity was further evaluated by experts in dentistry and environmental health, who reviewed the questionnaire items for relevance and clarity.

Responses to Likert-scale items were coded from 1 to 5, with higher scores representing more favorable EHL outcomes. Item responses within each domain were summed to obtain raw domain scores. The knowledge, attitude, confidence, and practice domain scores were reported using their original scoring ranges. The overall EHL score was converted to a standardized 0–100 scale using the following formula:

Standardized EHL score = (Observed score—Minimum possible score)/(Maximum possible score—Minimum possible score) × 100.

The standardized overall EHL score ranged from 0 to 100, with higher scores indicating higher levels of EHL. Participants with a standardized EHL score of ≥70 were categorized as having good EHL, whereas those with scores <70 were categorized as having poor EHL. The 70% threshold was investigator-defined and consistently applied as the predefined criterion for categorizing EHL levels.

### 2.1 Statistical analysis

Data was entered into Microsoft Excel and analyzed using R Statistical Software. Continuous variables were summarized using means and standard deviations (SD), whereas categorical variables were presented as frequencies and percentages. Normality assumptions were evaluated using the Kolmogorov–Smirnov test and Q–Q plots.

Comparisons between dentists and non-dentists were performed using chi-square tests or Fisher’s exact tests for categorical variables and independent t-tests for continuous variables. Spearman’s correlation analysis was conducted to assess relationships among literacy domains. There were no missing data for the variables included in the analyses. A standardized EHL score of ≥70 was predefined as the threshold for good EHL, whereas scores <70 were categorized as poor EHL. Multivariable logistic regression analyses were performed separately for dentists and non-dentists to identify factors independently associated with good EHL levels. All prespecified demographic and professional variables were entered simultaneously into the multivariable logistic regression models, which were performed separately for dentists and non-dentists. Adjusted odds ratios (aORs) with 95% confidence intervals (CIs) were reported. Statistical significance was established at *p* ≤ 0.05.

## 3 Results

### 3.1 Participant characteristics

A total of 384 participants were included in the study, comprising 258 dentists (67.2%) and 126 non-dentist oral healthcare professionals (32.8%). The required sample size was calculated *a priori* assuming a 95% confidence level, a 5% margin of error, and a conservative response distribution of 50%. Overall, 66.4% of participants were female, and nearly half (46.1%) were younger than 30 years. Significant differences were observed between the two groups regarding age distribution, gender, and educational level. Dentists were more frequently younger than 30 years, whereas non-dentists were more commonly aged 30–49 years (*p* < 0.010). Non-dentists demonstrated a higher proportion of female participants compared with dentists (77.0% vs. 61.2%, *p* < 0.010). Graduate-level education was also more prevalent among non-dentists (42.9% vs. 31.0%, *p* = 0.030) (Table 1). Regarding information sources, online platforms represented the most frequently reported source of environmental health information in both groups, whereas scientific journals and periodicals were less commonly utilized (Figure 1).

**Table 1:** Demographic characteristics of the study groups.

Variables	Levels	Dentist *n* (%)	Non-dentist *n* (%)	Total *n* (%)	*p*-value
Age	<30	133 (51.55)	44 (34.92)	177 (46.09)	<0.010
	30–49	108 (41.86)	62 (49.21)	170 (44.27)	
	50–65	17 (6.59)	20 (15.87)	37 (9.64)	
Gender	Female	158 (61.24)	97 (76.98)	255 (66.41)	<0.010
	Male	100 (38.76)	29 (23.02)	129 (33.59)	
Education	College degree (Bachelor’s Degree)	178 (68.99)	72 (57.14)	250 (65.10)	0.030
	Graduate degree (Master’s, Doctoral Degree)	80 (31.01)	54 (42.86)	134 (34.90)	
Residence	Riyadh	189 (73.26)	84 (66.67)	273 (71.09)	0.220
	Others	69 (26.74)	42 (33.33)	111 (28.91)	
Nationality	Saudi	196 (75.97)	96 (76.19)	292 (76.04)	1.000
	Others	62 (24.03)	30 (23.81)	92 (23.96)	

**Figure 1:**
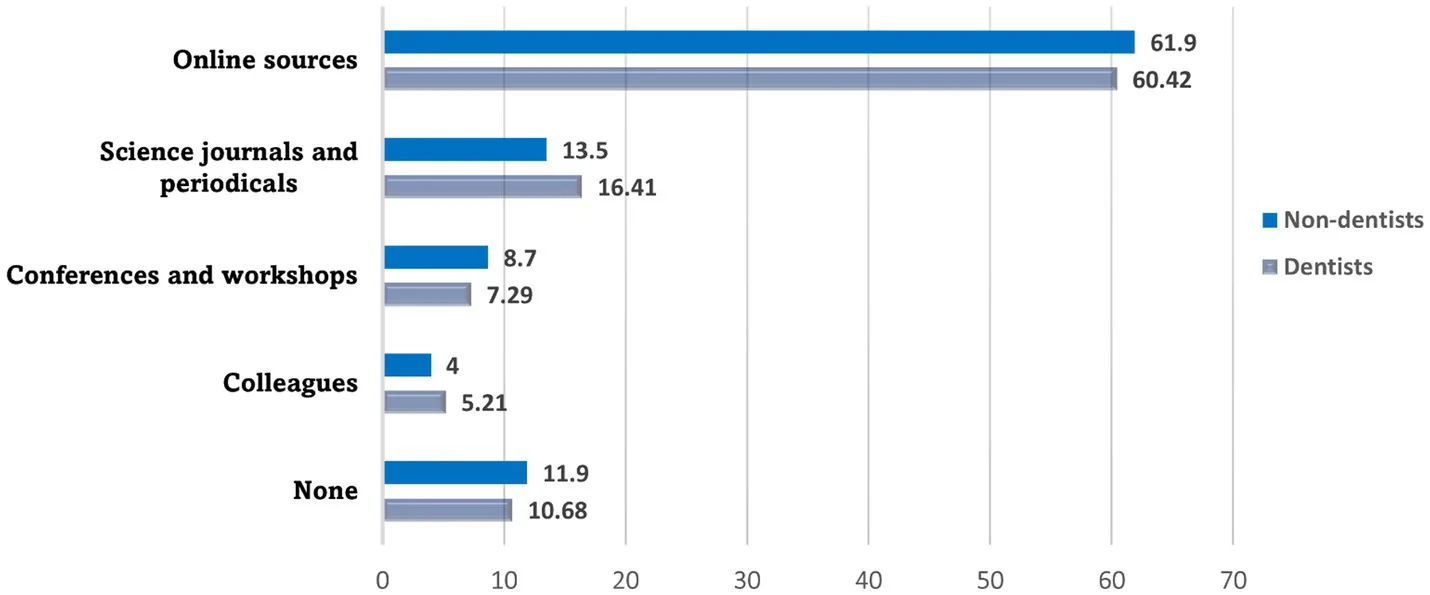
Sources of environmental health information among the study groups. Participants could select more than one source.

### 3.2 Environmental health literacy levels

The overall EHL score was comparable between dentists and non-dentists, with mean (SD) scores of 70.50 (15.11) and 73.38 (15.84), respectively (*p* = 0.090). Similarly, no statistically significant difference was observed in the proportion of participants demonstrating good overall literacy levels between dentists and non-dentists (48.1% vs. 57.9%, *p* = 0.090). However, significant differences were reported within the knowledge domain. Non-dentists demonstrated significantly higher knowledge scores than dentists (19.02 (3.40) vs. 17.93 (3.46), *p* < 0.010). Additionally, a greater proportion of non-dentists achieved good knowledge levels compared with dentists (64.3% vs. 51.6%, *p* = 0.020). No statistically significant differences were identified between the groups regarding attitude, confidence, or practice scores and levels (Figure 2 and Table 2).

**Figure 2:**
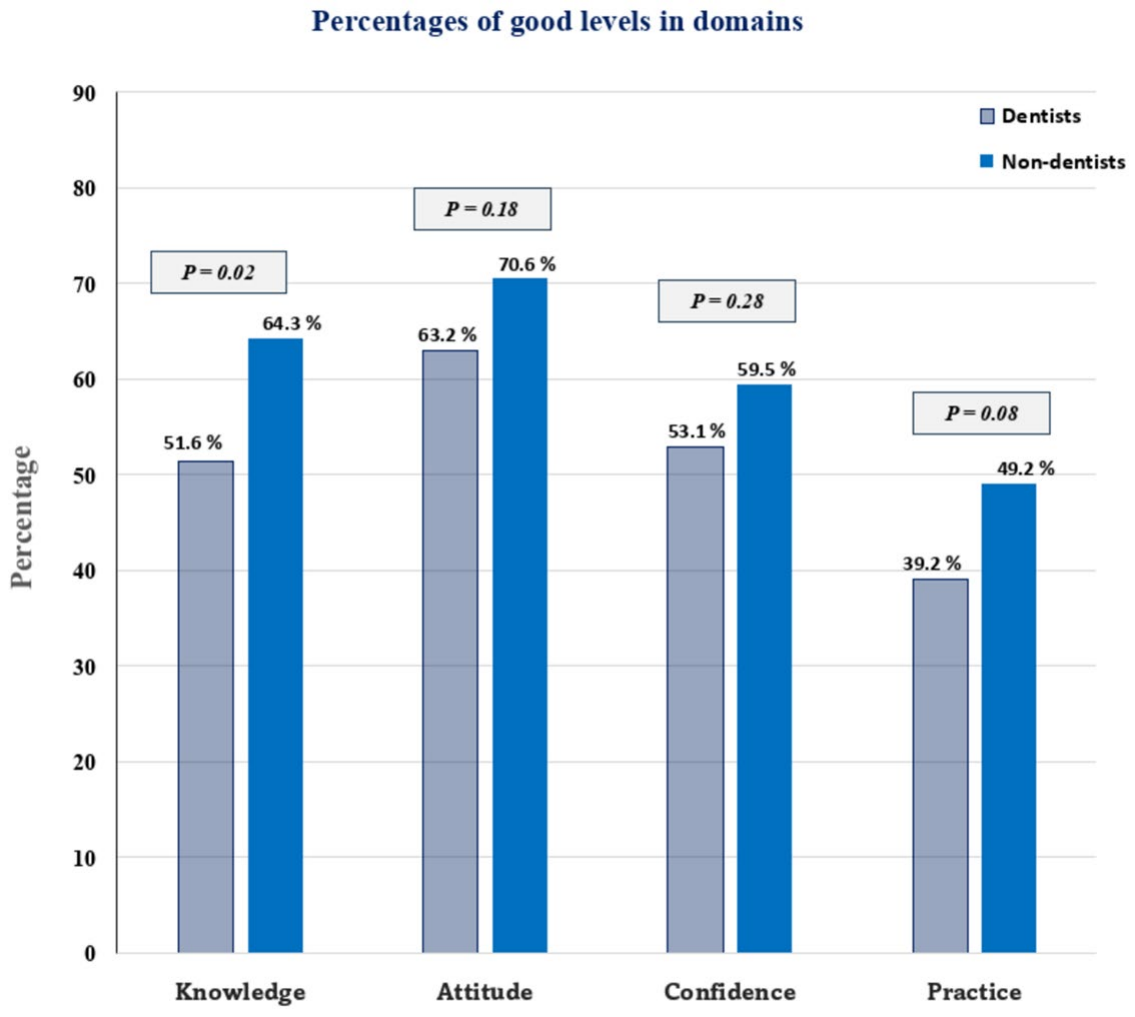
Percentage of participants with good environmental health literacy levels across survey domains in the study groups.

**Table 2:** Environmental health literacy scores and levels among the study groups.

Variables	Levels	Dentist *n* (%)	Non-dentist *n* (%)	Total *n* (%)	*p*-value
Literacy score	Mean (SD)	70.50 (15.11)	73.38 (15.84)	71.45 (15.39)	0.090
Literacy level	Good	124 (48.06)	73 (57.94)	197 (51.30)	0.090
	Poor	134 (51.94)	53 (42.06)	187 (48.70)	
Knowledge score	Mean (SD)	17.93 (3.46)	19.02 (3.40)	18.29 (3.48)	<0.010
Knowledge level	Good	133 (51.55)	81 (64.29)	214 (55.73)	0.020
	Poor	125 (48.45)	45 (35.71)	170 (44.27)	
Attitude score	Mean (SD)	22.10 (4.47)	22.87 (4.38)	22.35 (4.45)	0.110
Attitude level	Good	163 (63.18)	89 (70.63)	252 (65.62)	0.180
	Poor	95 (36.82)	37 (29.37)	132 (34.38)	
Confidence score	Mean (SD)	14.0 (3.78)	14.42 (3.90)	14.16 (3.82)	0.360
Confidence level	Good	137 (53.10)	75 (59.52)	212 (55.21)	0.280
	Poor	121 (46.90)	51 (40.48)	172 (44.79)	
Practice score	Mean (SD)	16.43 (4.85)	17.07 (5.60)	16.64 (5.11)	0.250
Practice level	Good	101 (39.15)	62 (49.21)	163 (42.45)	0.080
	Poor	157 (60.85)	64 (50.79)	221 (57.55)	

### 3.3 Correlation between literacy domains

Spearman’s correlation analysis demonstrated significant moderate-to-strong positive correlations among all EHL domains in both groups (all *p* < 0.010). Knowledge scores were positively associated with attitude, confidence, and practice scores, indicating that higher environmental health knowledge was consistently associated with more favorable perceptions and behaviors (Table 3).

**Table 3:** Correlations between environmental health literacy domains among the study groups.

Variable 1	Variable 2	Dentist group		Non-dentist group	
		coefficient	*p*-value	coefficient	*p*-value
Attitude	Practice	0.700	<0.010	0.820	<0.010
Attitude	Confidence	0.770	<0.010	0.780	<0.010
Knowledge	Attitude	0.780	<0.010	0.780	<0.010
Knowledge	Practice	0.740	<0.010	0.740	<0.010
Knowledge	Confidence	0.770	<0.010	0.780	<0.010
Practice	Confidence	0.800	<0.010	0.830	<0.010

### 3.4 Factors associated with good literacy levels

Among dentists, multivariable logistic regression analysis revealed that participants aged 30–49 years (aOR = 3.01, 95% CI: 1.42–6.56, *p* = 0.005) and 50–65 years (aOR = 4.28, 95% CI: 1.20–16.24, *p* = 0.027) had significantly greater odds of good literacy levels compared with participants younger than 30 years. Attendance at environmental health conferences or scientific events was strongly associated with good literacy levels among dentists (aOR = 4.02, 95% CI: 1.88–8.93, *p* < 0.001). Similarly, receiving formal environmental health training significantly increased the likelihood of good literacy (aOR = 2.35, 95% CI: 1.21–4.59, *p* = 0.012). Saudi nationality was associated with lower odds of good literacy levels (aOR = 0.28, 95% CI: 0.13–0.61, *p* = 0.001) (Table 4).

**Table 4:** Factors associated with good environmental health literacy among dentists.

Variables	Levels	Poor	Good	Crude OR (95%CI, *p*-value	Adjusted OR (95%CI, *p*-value
Age	30–49	40 (37.0)	68 (63.0)	3.22 (1.90–5.50, *p* < 0.001)	3.01 (1.42–6.56, *p* = 0.005)
	50–65	7 (41.2)	10 (58.8)	2.70 (0.97–7.89, *p* = 0.059)	4.28 (1.20–16.24, *p* = 0.027)
	<30	87 (65.4)	46 (34.6)	—	—
Gender	Female	89 (56.3)	69 (43.7)	—	—
	Male	45 (45.0)	55 (55.0)	1.58 (0.95–2.62, *p* = 0.077)	1.17 (0.63–2.16, *p* = 0.623)
Education	College degree	100 (56.2)	78 (43.8)	0.58 (0.34–0.98, *p* = 0.043)	1.14 (0.53–2.51, *p* = 0.733)
	Graduate degree	34 (42.5)	46 (57.5)	—	—
Residence	Others	24 (34.8)	45 (65.2)	—	—
	Riyadh	110 (58.2)	79 (41.8)	0.38 (0.21–0.67, *p* = 0.001)	0.66 (0.32–1.36, *p* = 0.258)
Nationality	Others	15 (24.2)	47 (75.8)	—	—
	Saudi	119 (60.7)	77 (39.3)	0.21 (0.11–0.39, *p* < 0.001)	0.28 (0.13–0.61, *p* = 0.001)
Previously received an environmental health formal training?	No	95 (63.8)	54 (36.2)	—	—
	Yes	39 (35.8)	70 (64.2)	3.16 (1.90–5.32, *p* < 0.001)	2.35 (1.21–4.59, *p* = 0.012)
Recently attended an environmental health conference or scientific event?	No	116 (61.1)	74 (38.9)	—	—
	Yes	18 (26.5)	50 (73.5)	4.35 (2.40–8.21, *p* < 0.001)	4.02 (1.88–8.93, *p* < 0.001)

—indicates the reference category.

Among non-dentists, attendance at environmental health scientific events remained the strongest predictor of good literacy levels (aOR = 12.25, 95% CI: 3.63–51.11, *p* < 0.001). Residence in Riyadh was independently associated with lower odds of good literacy (aOR = 0.33, 95% CI: 0.12–0.84, *p* = 0.023). Other demographic factors were not significantly associated with literacy levels after adjustment (Table 5).

**Table 5:** Factors associated with good environmental health literacy among non-dentists.

Variables	Levels	Poor	Good	Crude OR (95%CI, *p*-value	Adjusted OR (95%CI, *p*-value
Age	30–49	32 (51.6)	30 (48.4)	0.39 (0.17–0.88, *p* = 0.025)	0.82 (0.26–2.61, *p* = 0.738)
	50–65	8 (40.0)	12 (60.0)	0.63 (0.21–1.93, *p* = 0.411)	1.48 (0.26–8.05, *p* = 0.654)
	<30	13 (29.5)	31 (70.5)	—	—
Gender	Female	45 (46.4)	52 (53.6)	—	—
	Male	8 (27.6)	21 (72.4)	2.27 (0.95–5.92, *p* = 0.076)	1.17 (0.36–3.88, *p* = 0.794)
Education	College degree	24 (33.3)	48 (66.7)	2.32 (1.13–4.84, *p* = 0.023)	2.03 (0.73–5.70, *p* = 0.174)
	Graduate degree	29 (53.7)	25 (46.3)	—	—
Residence	Others	14 (33.3)	28 (66.7)	—	—
	Riyadh	39 (46.4)	45 (53.6)	0.58 (0.26–1.23, *p* = 0.162)	0.33 (0.12–0.84, *p* = 0.023)
Nationality	Others	10 (33.3)	20 (66.7)	—	—
	Saudi	43 (44.8)	53 (55.2)	0.62 (0.25–1.43, *p* = 0.269)	0.66 (0.20–2.12, *p* = 0.490)
Previously received an environmental health formal training?	No	43 (56.6)	33 (43.4)	—	—
	Yes	10 (20.0)	40 (80.0)	5.21 (2.35–12.44, *p* < 0.001)	1.87 (0.62–5.62, *p* = 0.262)
Recently attended an environmental health conference or scientific event?	No	49 (58.3)	35 (41.7)	—	—
	Yes	4 (9.5)	38 (90.5)	13.30 (4.81–47.41, p < 0.001)	12.25 (3.63–51.11, p < 0.001)

—indicates the reference category.

## 4 Discussion

The questionnaire used to collect the data in the current study was developed around four complementary domains (knowledge, attitudes, confidence, and practices) which were intended to capture different dimensions of an oral healthcare professional’s capacity to recognize, understand, evaluate, and respond to environmental health issues relevant to dental practice. Knowledge assesses understanding of environmental health concepts and hazards; attitudes assess perceptions and beliefs regarding the importance of environmental health; confidence assesses perceived ability to identify and address environmental health concerns; and practice assesses the extent to which environmental health principles are incorporated into professional activities. The number of individuals who received the invitation was not recorded; therefore, the response rate was not calculated. Overall, the findings demonstrated moderate environmental health literacy among participants, with approximately half of the respondents achieving good literacy levels. Although overall literacy scores did not significantly differ between dentists and non-dentists, important differences were identified within the knowledge domain, where non-dentists demonstrated significantly higher scores and proportions of good knowledge levels.

These findings suggest that there is room for strengthening environmental health literacy among oral healthcare professionals in the study population. Oral healthcare settings involve frequent exposure to biomedical waste, hazardous materials, chemical disinfectants, radiographic procedures, and environmentally sensitive clinical processes. Adequate EHL is therefore potentially important for supporting safe and sustainable clinical practice as well as effective patient counseling regarding environmental health risks (7, 8). However, the present study did not directly assess whether EHL translates into specific environmental or clinical outcomes; therefore, these potential implications should not be interpreted as effects demonstrated by this study.

In the current study, non-dentist participants demonstrated higher environmental health knowledge scores than dentists. One possible explanation for this finding is a greater exposure among academicians and allied healthcare professionals to interdisciplinary public health, environmental health, and sustainability initiatives. In contrast, dentists working primarily in clinical settings may have fewer opportunities to engage with environmental health education because of competing clinical or administrative responsibilities. Furthermore, the limited integration of environmental health and sustainability concepts into traditional dental curricula and professional development programs may contribute to lower knowledge levels among dental practitioners (11, 12).

A previous study by Chanioti et al. (13) found that although both dentists and dental students expressed positive attitudes toward environmental protection, dental students demonstrated greater environmental awareness and adoption of sustainable practices than practicing dentists. Christidis et al. (14) further demonstrated that dental and nursing professionals often felt inadequately prepared to address climate change and sustainability issues in healthcare with their patients. A later study by Sonmez et al. (15) showed that targeted educational interventions significantly improved climate–health literacy among dental students, suggesting that structured training can effectively strengthen environmental health competencies. Collectively, these findings suggest that integration of environmental health, climate change, and sustainability concepts into both healthcare curricula and continuing professional education to improve EHL among oral healthcare professionals. However, the effectiveness of such interventions among practicing oral healthcare professionals requires further investigation.

The present study demonstrated significant positive correlations among all EHL domains, including knowledge, attitudes, confidence, and practices, suggesting that these components are interconnected rather than independent. However, because of the cross-sectional design, these associations should not be interpreted as evidence that one domain causes or leads to another. The observed relationships support multidimensional health literacy frameworks, which propose that knowledge serves as a foundation for developing favorable attitudes, self-efficacy, and the adoption of health-promoting behaviors (16–18). Consistent with this interpretation, attendance at environmental health conferences or scientific events was the strongest predictor of adequate EHL in both professional groups, while formal environmental health training was independently associated with higher literacy among dentists. These findings identify educational exposure as a potentially important correlate of EHL; however, the cross-sectional design does not allow us to determine whether participation in environmental health education increases EHL or whether individuals with greater interest or pre-existing EHL are more likely to seek such educational opportunities.

Among non-dentists, the adjusted estimate had a wide confidence interval, indicating limited precision and warranting cautious interpretation given the small number of participants classified as having poor EHL. The non-dentist group also included academicians, dental assistants, and allied health professionals; therefore, the observed knowledge difference may not be uniform across these subgroups. It is also important to note that the absence of significant differences in attitude, confidence, and practice despite the knowledge difference suggests that greater knowledge alone may not necessarily correspond to differences in perceptions, confidence, or reported practices. These domains may also be influenced by professional context and other factors not assessed in the present study.

Older dentists demonstrated higher EHL, which could potentially reflect differences in professional experience, continuing professional development, or cumulative exposure to environmental and occupational health information over the course of a career. However, these potential mechanisms were not directly assessed in this study, and the observed association should therefore not be interpreted as evidence that professional experience or continuing education caused higher EHL. This is consistent with evidence that structured educational interventions can improve EHL and related competencies (19, 20). A study by Tomsho et al. (19) reported significant gains in professionals’ environmental exposure knowledge and overall EHL following a virtual intervention while O’Brien et al. (20) found that health literacy–focused training consistently enhances the application of health literacy principles in clinical practice. The observed age gradient in EHL may potentially be related to differences in educational exposure or professional experience; however, this hypothesis requires further investigation. Longitudinal and interventional studies are needed to determine whether cumulative educational exposure or other professional factors contribute to differences in EHL across age groups.

Limitations of the current study include the convenience sampling approach, which may have introduced selection bias because participation was voluntary and individuals who were more interested in environmental health or sustainability may have been more likely to participate. Consequently, the participants may not fully represent the broader population of oral healthcare professionals in Saudi Arabia. Therefore, the findings should be interpreted with caution, and their generalizability to oral healthcare professionals in other settings or to the wider population is limited. Although the questionnaire underwent pilot testing and expert review for content validity, quantitative internal-consistency reliability as well as construct validity were not assessed in the present study. Future studies using probability-based or more diverse sampling approaches are warranted to improve representativeness and generalizability.

The findings of this study have important implications for dental education, healthcare institutions, and policymakers. Environmental health and sustainability principles should be systematically integrated into dental curricula, continuing professional development, and institutional policies. Interprofessional education may further strengthen environmental literacy by fostering collaboration between dentists, public health professionals, and allied healthcare disciplines. In addition, healthcare organizations should actively support participation in scientific meetings and sustainability-focused training to promote environmentally responsible clinical practice.

## 5 Conclusion

EHL among oral healthcare professionals was moderate, with substantial proportions demonstrating poor literacy. Non-dentists showed higher environmental health knowledge, while attendance at scientific events and formal training were associated with higher EHL levels. These findings suggest the potential value of integrating environmental health education into dental curricula and continuing professional development to strengthen environmentally responsible practice and support sustainable healthcare systems.

## Data Availability

The original contributions presented in the study are included in the article/Supplementary material, further inquiries can be directed to the corresponding author.
