# Supplementary material for: Environmental health literacy among oral healthcare professionals—a comparison across professional groups
Source: Front Public Health. 2026 Oct 19;14:1933350. doi: 10.3389/fpubh.2026.1933350 (PMC13724372; doi:10.3389/fpubh.2026.1933350)
Supplement: Supplementary file 1 [file Table_1.docx]

**Questionnaire form**

The general health condition of any population depends on the environment and the surrounding ecosystem. According to the most recent WHO data, it is estimated that 1 in 4 people, globally, dies due to preventable environmental health risks. Climate change, for example, already has detrimental effects on health and these impacts are likely to increase significantly in the future. In an effort to explore the current environmental health literacy standards of dental practitioners, we kindly ask you to participate in this short survey. Answers will be collected anonymously and stored securely. Data will be solely utilized for research purposes including guiding future community–academic partnerships in environmental health programs.

**Questions**

- Age (Y) ˂ 30

30-49

50-65

> 65

- Gender	Femal , Male
- Education College degree (Bachelor’s Degree)

Graduate degree (Master’s Degree, Doctoral Degree)

- Residence Riyadh

Other

- Nationality Saudi Arabia, Other
- What is your primary professional role?
  - Dentist/clinical practice
  - Dental academician/researcher
  - Allied health professional
  - Other
- Speciality (for practitioners)
  - Advanced General Dentistry
  - Dental Assistant
  - Dental Hygienist
  - Dental Public Health
  - Dental Radiologist
  - Endodontist
  - General Dentist
  - Oral Surgeon
  - Oral Medicine
  - Orthodontist
  - Periodontist
  - Prosthodontist
  - Paediatric dentist
  - Restorative Dentist
  - Other
- I have previously received an environmental health formal training
  - Yes
  - No
- I have recently attended an environmental health conference or scientific event
  - Yes
  - No
- I routinely ask my patients about their exposure to environmental hazards.
  - Always
  - Often
  - Sometimes
  - Hardly ever
  - Never
- I consistently stay informed about environmental health risks, such as climate change and its potential impacts on public health.
  - Always
  - Often
  - Sometimes
  - Hardly ever
  - Never
- Where do you primarily get information on environmental health?
  - Science journals and periodicals
  - Online resources
  - Conferences and workshops
  - Colleagues
  - None
- How strongly do you agree with the following statement? “As a leading emitter of environmental pollutants, dental healthcare facilities significantly contribute to pollution and climate change”.
  - Strongly Agree
  - Agree
  - Neutral
  - Disagree
  - Strongly Disagree
- How strongly do you agree with the following statement? "Dental healthcare professionals are disproportionally affected by environmental risks”.
  - Strongly Agree
  - Agree
  - Neutral
  - Disagree
  - Strongly Disagree
- How strongly do you agree with the following statement? "I have a primary responsibility to address environmental health issues with my patients".
  - Strongly Agree
  - Agree
  - Neutral
  - Disagree
  - Strongly Disagree
- How strongly do you agree with the following statement? “One of the major attributes to an environmentally sustainable dental practice is the provision of a quality preventive care that drives less interventions and waste production”.
  - Strongly Agree
  - Agree
  - Neutral
  - Disagree
  - Strongly Disagree
- My workplace incorporates ecofriendly practices such as reduced energy consumption, renewable energy sources, and the effective use of water.
  - Always
  - Often
  - Sometimes
  - Hardly ever
  - Never
- I feel that recently patients are increasingly concerned about their exposure to environmental hazards, such as pollution and poor air quality.
  - Always
  - Often
  - Sometimes
  - Hardly ever
  - Never
- I believe that dental healthcare professionals should educate patients on the health risks of climate change.
  - Strongly Agree
  - Agree
  - Neutral
  - Disagree
  - Strongly Disagree
- How confident are you in your ability to discuss environmental health risks with your patients?
  - Very Confident
  - Confident
  - Neutral
  - Not Confident
  - Not Confident at All
- I am aware of the environmental impact concerns related to the most commonly used dental restorative materials such as amalgam and mercury release as well as the microplastic pollution linked to the resin-based composite.
  - Strongly Agree
  - Agree
  - Neutral
  - Disagree
  - Strongly Disagree
- I incorporate environmental sustainability strategies, such as remote clinical consultations and teledentistry when possible, in my practice to reduce patients travel, mitigate air pollution, and contribute to the Net Zero carbon emissions target by 2040 and similar initiatives.
  - Always
  - Often
  - Sometimes
  - Hardly ever
  - Never
- I feel trained and confident in environmental history taking for patients who have special vulnerabilities to environmental exposures such as pregnant females.
  - Very Confident
  - Confident
  - Neutral
  - Not Confident
  - Not Confident at All
- I believe that the current growing body of evidence linking children’s mental health to environmental pollutants is reliable.
  - Strongly Agree
  - Agree
  - Neutral
  - Disagree
  - Strongly Disagree
- I believe that routine guidance to patients on the health effects of environmental exposures is a high priority.
  - Strongly Agree
  - Agree
  - Neutral
  - Disagree
  - Strongly Disagree
- I feel confident advising patients on how to reduce their risk of exposure to potential contaminants such as pesticides and heavy metals.
  - Very Confident
  - Confident
  - Neutral
  - Not Confident
  - Not Confident at All
- I have recently changed my behaviour in some way to reduce my exposure to environmental hazards.
  - Strongly Agree
  - Agree
  - Neutral
  - Disagree
  - Strongly Disagree
- My workplace provides the necessary resources to stay updated on environmental health literacy.
  - Always
  - Often
  - Sometimes
  - Hardly ever
  - Never
- I do what is necessary to ensure my home is free of toxicants, such as lead and radon.
  - Always
  - Often
  - Sometimes
  - Hardly ever

Never

- I feel confident in my self-efficacy in managing potential environmental hazards at my home and workplace.
  - Very Confident
  - Confident
  - Neutral
  - Not Confident
  - Not Confident at All
- How interested are you in participating in further training on environmental health?

- Very Interested

- Interested

- Neutral

- Not Interested

- Not Interested at All
